# Supplementary material for: Mitochondrial DNA mosaicism in normal human somatic cells
Source: Nat Genet. 2024 Jul 22;56(8):1665–77. doi: 10.1038/s41588-024-01838-z (PMC11319206; doi:10.1038/s41588-024-01838-z)
Supplement: Supplementary file 1 — Supplementary Notes 1–6 and Supplementary Figs. 1–4. [file 41588_2024_1838_MOESM1_ESM.pdf]

nature genetics

Supplementary information
https://doi.org/10.1038/s41588-024-01838-z

Mitochondrial DNA mosaicism in normal human somatic cells

In the format provided by the authors and unedited

Supplementary Information for:

Mitochondrial DNA mosaicism in normal human somatic cells

Jisong An, Chang Hyun Nam, Ryul Kim, Yunah Lee, Hyein Won, Seongyeol Park, Won Hee Lee, Hansol Park, Christopher J. Yoon, Yohan An, Jie-Hyun Kim, Jong Kwan Jun, Jeong Mo Bae, Eui-Cheol Shin, Bun Kim, Yong Jun Cha, Hyun Woo Kwon, Ji Won Oh, Jee Yoon Park, Min Jung Kim, and Young Seok Ju

Table of contents

Supplementary Note 1. MRCA of the clonal phylogenetic tree 2

Supplementary Note 2. Potential culture-associated events in the clones 2

Supplementary Note 3. Validation of caVAF 4

Supplementary Note 4. Het_{FE} variants in family data 6

Supplementary Note 5. Mitochondrial turnover models 6

Supplementary Note 6. mtDNA postzygotic recurrent mutations 8

Supplementary Figures 1-4 10

Supplementary Table Legends 15

Supplementary References 16

Supplementary Notes

Supplementary Note 1. MRCA of the clonal phylogenetic tree

In this study, early phylogenetic trees were reconstructed using somatic mutations in the nuclear genome identified in whole-genome sequences (WGSs) of multiple clones for each individual. Since we obtained clones from only one type of tissue per individual, it was essential to precisely identify the most recent common ancestor (MRCA) cell depicted in the constructed phylogenetic tree.

To this end, we tracked lineage-defining somatic mutations in the matched bulk blood tissues per individual. Bulk blood tissue is a collection of multiple cells and a tissue type different from clones (colorectal epithelium or fibroblast), so it could provide insights into the timing of MRCA occurrence. First, we assessed whether lineage-defining mutations of the early branches were detected in the matched bulk blood and then calculated their variant allele frequencies (VAFs) in the bulk blood. Then, the averaged VAFs of mutations in the first two earliest branches (Lineages 1 and 2) were calculated.

We found that somatic mutations specific to the initial branches of all phylogenetic trees were also identified in the matched bulk blood tissues (**Extended Data Fig. 2a**), indicating that the cellular branching had occurred before cell fate determination or gastrulation. This suggests that the phylogenetic tree can represent early embryogenesis. Moreover, merging the VAFs of lineage-defining mutations from Lineages 1 and 2 shows that they were typically present at approximately 50% in most individuals (**Extended Data Fig. 2b**), implying that these two lineages collectively comprised entire blood cells. This indicates that Lineage 1 and Lineage 2 represent lineages corresponding to the 2-cell stage of early embryogenesis, and the MRCA cell is equivalent and/or close to the fertilized egg, as previously reported[1-4]. Therefore, when the same mitochondrial DNA (mtDNA) variant appears repeatedly in multiple clones derived from a particular donor and their MRCA cell is the first node of the clonal phylogeny, we can conclude that the origin of the variant is likely the fertilized egg.

Supplementary Note 2. Potential culture-associated events in the clones

We conducted cell culture techniques to obtain clones derived from single cells. To recognize the potential for artifacts due to cell culture, we performed serial culture on the same clone. Then, we assessed the rate of culture-associated events and changes in VAF during the

culture, as previously reported[3,4]. Initially, we isolated single cells (or crypts) for clonal expansion to obtain single-cell-derived clones, referred to as mother clones, which were used for our analysis. Subsequently, we cultured the mother clones and performed single-cell isolation to carry out a second clonalization, which we called daughter clones. We then conducted WGS on both mother and daughter clones and compared them. The culture period before the clonal expansion of the mother clones was approximately 27 days on average. The additional culture period until single-cell isolation of the daughter clones was approximately 73.5 days. Therefore, we observed changes during the 73.5-day culture using the daughter clones. We obtained 47 pairs of mother–daughter clones from 10 mother clones (**Extended Data Fig. 3a**). The clones were composed of colon organoids, fibroblast clones, and in-house stomach organoids[3,4].

From 47 pairs of WGSs obtained from mother and daughter clones, we found 26 new mtDNA mutations, specifically in daughter clones. This enabled us to calculate the culture-associated mtDNA mutation rate: 26 mtDNA mutations / 47 clones / 73.5 days = 0.0075 per clone per day. By applying this rate, we estimated the maximum culture-associated mtDNA mutations in the mother clones, which is 0.0075 per clone per day × 27 days = 0.20 mutations, assuming rapid clonal expansion on the 27th day and accumulation of culture-associated mutations until that point. This number suggests that the highest proportion of the culture-associated mtDNA mutations in identified mtDNA variants is 6.5%, calculated as 0.20/3.1×100 (where 3.1 denotes the average number of mosaic mtDNA variants per clone). Of note, most of the newly identified mutations in the daughter clones may originate from the mother clones (real mutations below the detection threshold), expanded during culture to become detectable.

Then, we compared VAFs of 49 mtDNA mutations, which were detectable in both mother and daughter clones, to assess the impact of cell culture on VAF. The median values of the VAFs observed in daughter clones showed a robust positive correlation with the VAFs in mother clones and were nearly identical in value (**Extended Data Fig. 3b**). Moreover, direct comparisons of the VAFs between mother and daughter clones for each variant revealed that the VAFs in daughter clones were distributed near those of the mother clones, with minimal differences (**Extended Data Fig. 3c**). This indicates that the VAFs remained relatively stable during the approximately 73.5-day culture process. Although some clones showed variations in VAF, these changes were not extreme and were not expected to impact the overall analysis results significantly. VAF changes in our main data could be less than observed in the serial cultures, as the culture period is likely shorter than half the duration.

Furthermore, we aimed to assess whether there were differences in the analysis results

between clones obtained through cell culture and those obtained without cell culture from the same tissue. To this end, we compared the mutational profiles of 431 colonic clones obtained from single-crypt-derived organoids with 432 patches from colon crypts obtained via laser capture microdissection (LCM)[5]. Colon crypts are known to be mono-clonal or at least dominant clonal because only a few stem cells compete with each other over time[6,7]. The LCM data was obtained by extracting DNA from tissues and sequencing without cell culture. The results revealed that the colon data obtained from organoids and LCM showed similar patterns in 1) the pattern of S_{VAF} (the sum of clone-VAFs of all detected postzygotic simple (PZ_{simple}) mutations in a clone) increasing with age (**Extended Data Fig. 3d**), 2) mutational spectrum in mtDNA (**Extended Data Fig. 3e**) and 3) the proportion of mutations classified based on functional consequences, including synonymous, missense, and truncating mutations (**Extended Data Fig. 3f**). The clone-VAF distribution was also similar between organoids and LCM samples, except for the absence of 100% clone-VAF in LCM samples because LCM samples were not 100% clonal (**Extended Data Fig. 3g**). The presence or absence of cell culture does not alter the mutational profile trends observed in colon data. This indicates that the cell culture does not significantly change the VAF of mtDNA or induce selective pressure on specific types of variants. It also demonstrates that our study did not investigate selectively cultured cells but rather the overall colorectal epithelial cell population.

In summary, our findings underscored the minimal impact of cell culture on mutational profiles and VAF. While cell culture may introduce some new variants and lead to slight changes in VAF, these alterations represent a negligible fraction of the overall discovered variants and lead to very low VAF. The minimal changes in VAF make it challenging for these alterations to affect our analysis results. Importantly, no discernible differences were observed when comparing clones generated with and without culture; the profiles were nearly identical. Therefore, although artifacts may occur during the culture process, they are not prevalent and unlikely to significantly alter the overall mutational landscape trends.

Supplementary Note 3. Validation of caVAF

We calculated the clone-averaged VAF (caVAF) for each mtDNA variant originating from the fertilized egg (Het_{FE}) by averaging the clone-VAF of all clones derived from the individual (**Extended Data Fig. 4c**). Interestingly, the calculated caVAFs of Het_{FE} variants were remarkably similar to their VAFs in the matched bulk blood (blood-VAF; **Fig. 2e**). Of note, the two VAFs (caVAF and blood-VAF) were measured by applying different methods to different cell types; thus, these two values are independent. The most plausible speculation for the

strong correlation between caVAF and blood-VAF is that these values represent the initial heteroplasmy level in the fertilized egg. We validated our speculation in two ways: computational simulation and analysis of monozygotic twins.

According to the Hardy–Weinberg equilibrium, the frequency of a variant in the nuclear genome remains constant across generations in the entire population. Likewise, we speculate that the initial VAF of a Het_{FE} variant is stable among a population of somatic cells. Therefore, the average VAF of the Het_{FE} variant calculated from tens to hundreds of clones of an individual (caVAF) should represent the VAF of the cell population; thus, the caVAF is expected to resemble the VAF in the fertilized egg. As the VAF from the bulk blood tissues is an averaged VAF of a Het_{FE} variant among many polyclonal blood cells, it should also be close to the initial heteroplasmy level in the fertilized egg.

To assess this speculation, we conducted computational simulations. We set a specific variant to exist in 10,000 cells at a particular initial VAF and then made it undergo multiple mitotic turnovers. Subsequently, the average VAF of 100 cells sampled from the entire pool of 10,000 cells was examined (caVAF). The initial VAF in the first cell represents the heteroplasmy level in the fertilized egg, and the 100 sampled cells represent the sequenced clones. The calculated caVAF remained similar to the initial VAF in the first cell despite multiple turnovers (**Extended Data Fig. 4d**). This indicates that the VAF of the Het_{FE} variant in the fertilized egg remains similar to the caVAF calculated from tens to hundreds of clones in somatic lineages.

Further, we analyzed bulk tissues of monozygotic twins and compared VAFs of Het_{FE} variants among them. We found 16 variants shared in the buccal swab tissues of 19 monozygotic twin siblings, most likely Het_{FE} variants because monozygotic twins come from the same fertilized egg. Indeed, the VAFs of Het_{FE} variants strongly correlated with each other (**Extended Data Fig. 4e**). These variants were also found in bulk blood tissues of the twins, and the VAFs between the buccal swab and bulk blood tissues were very similar (**Extended Data Fig. 4f**). This finding suggests that the VAFs of these variants in bulk tissues correspond to those in the fertilized egg of the twin.

Collectively, we found that the caVAF calculated from the VAFs of clones is similar to the VAF of the variant in the bulk tissues, as well as the original heteroplasmy level in the fertilized egg. It indicates that the initial level of a Het_{FE} variant maintains a stable VAF, on average, among a collection of polyclonal cells (blood or buccal tissues or a collection of multiple clones). Therefore, we considered the caVAF as a proxy for the heteroplasmy level of Het_{FE} variants in the fertilized egg and used it for subsequent analyses.

Supplementary Note 4. Het_{FE} variants in family data

We dissected the maternal origins of Het_{FE} variants using the family data (*n* = 294 families)[8]. We analyzed only variants with VAFs greater than 0.5% in the mother's and offspring's bulk tissues. Therefore, we considered all identified variants to be Het_{FE} variants shared by a considerable proportion of cells.

Interestingly, approximately 20% of Het_{FE} variants in the offspring were also detected as heteroplasmic variants in the mother's bulk blood (*n* = 82; **Fig. 2g** and **Extended Data Fig. 4g**), strongly indicating that these variants were pan-body mosaic in the mother and were not confined to the maternal germline; thus, they were present in the mother's first cell (fertilized egg) inherited from the maternal grandmother. The remaining 80% of Het_{FE} variants in the offspring were not found in the mother's bulk blood and were likely acquired during the mother's oogenesis. Notably, Het_{FE} variants with high VAF in the offspring were more likely to be found in the mother's bulk blood (**Fig. 2g**). This indicates that Het_{FE} variants with high VAF in the offspring are likely to be Het_{FE} variants in the mother.

Furthermore, we traced the transmission of Het_{FE} variants to the next generation using all heteroplasmic variants in the mothers' blood tissues, which are most likely Het_{FE} variants in mothers (*n* = 369 from 407 mother–offspring pairs). Overall, ~20% of Het_{FE} variants were found in the next generation (**Fig. 2h**).

Collectively, we confirmed that 20% of the mother's Het_{FE} variants were transmitted to the offspring, and 20% of the offspring's Het_{FE} variants were inherited from the mother's fertilized egg (**Fig. 2h**). This suggests that despite the purification process during oogenesis, a substantial proportion of Het_{FE} variants are passed on the next generation.

Supplementary Note 5. Mitochondrial turnover models

Considering the nature of mitochondria, we developed two turnover models: the mitotic and homeostatic turnover models (**Fig. 3c**; **Extended Data Fig. 5b–e**). These models were devised to reflect mitochondrial turnover regardless of cell division. In the mitotic turnover model, representing cell-cycle-dependent mitochondrial turnover, mtDNA is duplicated and randomly segregated into two daughter cells during cell division. The homeostatic turnover model reflects cell-cycle-independent mitochondrial turnover, where a damaged mtDNA is promptly replaced by one event of mtDNA replication.

In both models, a *turnover* is defined as the replication of mtDNA for n times, where the basal mtDNA copy number in a somatic cell is n, resulting in a 100% refreshment of the mtDNA population. In the mitotic turnover model, all mtDNA copies in a cell have an equal chance of being duplicated. Thus, mtDNA copies increase one by one until they reach twice the basal copy number, after which they are randomly segregated into daughter cells due to mitosis (**Extended Data Fig. 5b**). Hence, in this model, when the basal mtDNA copy number in a single cell is denoted as n, the mtDNA copy number gradually increases until it reaches $2n$, then decreases back to n (**Extended Data Fig. 5c**). In the homeostatic turnover model, random replication occurs whenever one mtDNA copy undergoes degradation for n times (**Extended Data Fig. 5d**). Consequently, the mtDNA copy number in a cell is maintained at the basal level (n; **Extended Data Fig. 5e**). These two models describe two extreme situations; hence, the actual mitochondrial turnover is expected to occur as a mixture of both.

Comparing the simulation results of the two turnover models revealed that the homeostatic turnover model showed a more pronounced drift effect than the mitotic turnover model. Consequently, turnover rates inferred from the homeostatic turnover model were 50% lower than those inferred from the mitotic turnover model (**Fig. 3h** and **Supplementary Fig. 1**). However, this outcome can be attributed to the discrepancy in two factors influencing drift: 1) the average mtDNA copy number and 2) the number of mtDNA replications.

In both turnover models, we initially set the same basal mtDNA copy number. However, due to the intricacies of the model structures, the average number of mtDNA copies within a cell during one turnover differs. In the mitotic turnover model, mtDNA copies gradually increase from the basal mtDNA copy number (n) until doubling before mitosis occurs. This results in an average mtDNA copy number in a cell of $1.5n$ during one mitotic turnover. In contrast, the homeostatic turnover model maintains a constant basal mtDNA copy number, resulting in an average mtDNA copy number of n during one homeostatic turnover. Therefore, when setting the same basal mtDNA copy number in both the mitotic and homeostatic turnover models, the number of mtDNA replications remains the same. However, the average mtDNA copy number differs between the two models. The increase in mtDNA copy number weakens the effect of lifetime drift (**Extended Data Figs. 8** and **9**), necessitating more turnovers to achieve the same outcome. Therefore, to present similar results of drift, the mitotic turnover model requires more turnovers than the homeostatic turnover model.

If different basal mtDNA copy numbers are set for the two turnover models to achieve the same average mtDNA copy number, the number of mtDNA replications required for one turnover varies depending on the turnover definition. For example, if the basal mtDNA copy number is set to n in

the mitotic turnover model and 1.5*n* in the homeostatic turnover model, the mitotic turnover model requires random mtDNA replication for *n* times, whereas the homeostatic turnover model requires replication for 1.5*n* times. As a result, the replication count differs, altering the drift effect and making it challenging to attain consistent simulation outcomes across turnover models.

For these reasons, the absolute values estimated from the two turnover models, including 1) the average turnover count required for a variant with a specific caVAF to reach homoplasmy (**Fig. 3g**) and 2) the turnover rate per year determined by Het_{FE} variants (**Fig. 3h**), showed approximately a twofold difference (homeostatic turnover rate = 0.496×mitotic turnover rate; **Supplementary Fig. 1**). Of note, as previously mentioned, since the two turnover models represent extreme scenarios, the actual turnover rate should fall between these values, determined by the relative ratios of the two models for each cell type.

However, apart from the absolute turnover value, the overall trends in the simulation results remained similar irrespective of the turnover model. Additionally, the absolute mtDNA mutation rates were consistent across cell types and turnover models (5.0×10^{-8} per bp replication; **Fig. 4f**). When the basal mtDNA copy number was set to 750, the patterns of mtDNA and mtDNA heteroplasmy changes modeled in both the mitotic and homeostatic turnover models were also similar (**Fig. 7b, c**; **Supplementary Fig. 2**). Indeed, the tendency of a decrease in the extent of lifetime drift as the basal mtDNA copy number increases was consistently observed in both turnover models (**Extended Data Figs. 8** and **9**; **Supplementary Fig. 3**).

In summary, although the absolute estimated values from the two models differed, we underscored the robustness of the overall model of mtDNA dynamics. Moreover, we presented values from the homeostatic turnover model parallel to the mitotic turnover model, emphasizing that both models demonstrate analogous lifetime drift effects and likely depict mitochondrial turnover.

Supplementary Note 6. mtDNA postzygotic recurrent mutations

Among 6,042 postzygotic mtDNA mutations, we categorized 390 variants in 32 mtDNA loci (6.5% of postzygotic mutations) as postzygotic recurrent ($PZ_{recurrent}$) mutations. $PZ_{recurrent}$ mutations are observed recurrently across multiple clones within a particular donor, similar to Het_{FE} variants. However, unlike Het_{FE} variants, $PZ_{recurrent}$ mutations are not confined to a single individual but are observed across multiple individuals (**Supplementary Fig. 4a, b**). Therefore, we consider $PZ_{recurrent}$ mutations to be recurrent mutations acquired independently multiple times in various clones within an individual rather than mutations acquired once in the MRCA cell.

Consistent with independent multiple acquisitions, specific $PZ_{recurrent}$ mutations often demonstrate an inconsistent phasing pattern with other variants (**Supplementary Fig. 4c**). For instance, we observed two variants in the vicinity of each other, m.12,417 CA>C and m.15,623 G>A, which are a $PZ_{recurrent}$ mutation and a PZ_{simple} mutation, respectively (**Supplementary Fig. 4a**). According to the pigeonhole principle, these two variants can be phased by sequencing reads. We then observed four different allele combinations: wild-type–wild-type, wild-type–mutant, mutant–wild-type, and mutant–mutant (**Supplementary Fig. 4c**). Application of the four-gamete test indicates the potential for 1) repeated mutation and 2) recombination between the two loci. Considering the low likelihood of mtDNA recombination and our observation of m.12,417 CA>C in multiple clones of DB6 and other individuals, the most plausible scenario for the four gametes involves m.12,417 CA>C as the repeated mutation. The phasing pattern of the four gametes further supports the fact that the classified $PZ_{recurrent}$ mutations occur recurrently and independently.

The identified $PZ_{recurrent}$ mutations were predominantly located in the hypervariable regions of the D-loop, homopolymer sequences, or both (**Extended Data Fig. 6a**). As expected, most $PZ_{recurrent}$ mutations in homopolymeric sequences were short insertions and deletions (InDels). These were unlikely to be sequencing errors, as VAFs of these mutations in mutant clones were considerably higher than background noise (**Supplementary Fig. 4d**). Furthermore, we explored the similarity of these $PZ_{recurrent}$ mutations to six hotspot mutations found in cancers[9]. Among these, four homopolymeric hotspots in Complex I genes were included in our $PZ_{recurrent}$ mutation list (m.10,947-10,952, m.11,032-11,038, m.11,867-11,872, m.12,418-12,415). These four $PZ_{recurrent}$ mutations consisted of short InDels within the homopolymeric sequences, suggesting their independent acquisition via DNA polymerase errors.

Notably, one $PZ_{recurrent}$ mutation, m.414 T>G, was exclusively detected in multiple fibroblast clones (**Supplementary Fig. 4e**). Given its association with cumulative exposure to ultraviolet (UV) light (measured by SBS7 mutation counts in the nuclear genome of a clone), we speculate its UV-dependent acquisition, as previously reported[10,11] (**Extended Data Fig. 6b**). Furthermore, despite cell type specificity, 13 loci exhibited a positive correlation with the age of individuals, suggesting that the loci were cumulatively acquired through mtDNA replication during their lifetime (**Supplementary Fig. 4f**).

Although these $PZ_{recurrent}$ mutations are indeed postzygotic-acquired, their independent and recurrent occurrences render them unsuitable for tracking heteroplasmy changes over time. Consequently, in subsequent analyses, we primarily utilized PZ_{simple} mutations.

Supplementary Figures

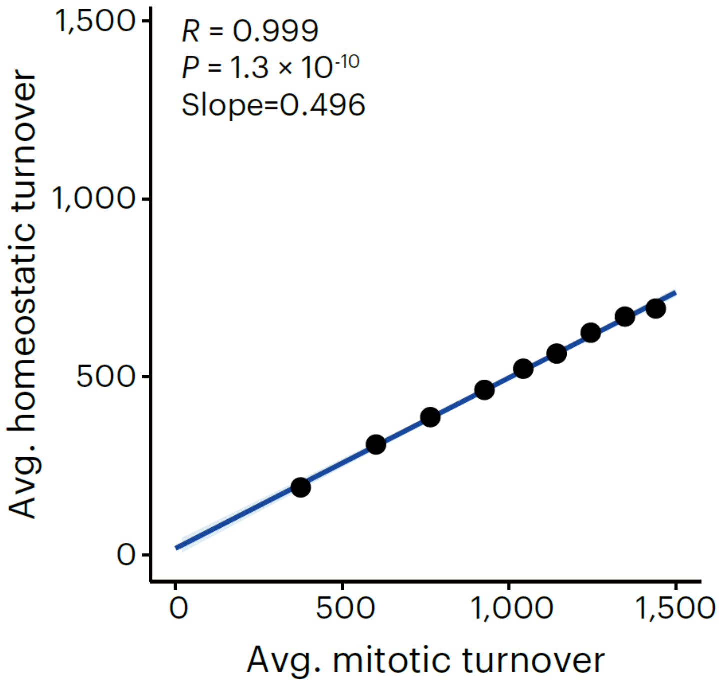

Supplementary Fig 1. Comparison between mitotic and homeostatic turnover model.

A scatter plot comparing the average turnover counts to reach homoplasmy in mitotic and homeostatic turnover models for nine caVAF values (0.1-0.9). The blue line and the shaded area indicate the regression line and its 95% confidence interval. The regression equation was calculated as y=0.496x through univariable linear regression analysis. The Pearson's correlation coefficient and *P* value are provided. Two-sided Pearson's correlation.

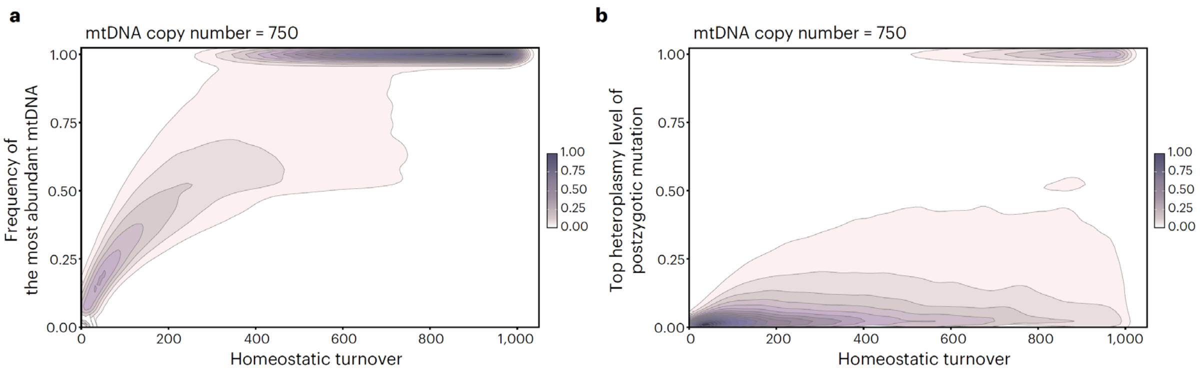

Supplementary Fig 2. Model for mtDNA dynamics with homeostatic turnover.

a, A contour plot representing how an mtDNA population changes with continuous homeostatic turnover from simulation studies assuming a baseline mtDNA copy number of 750. The *x* axis shows the homeostatic turnover count and the *y* axis shows the frequency of the most prevalent mtDNA, regardless of mutations. **c**, A contour plot representing how clone-VAF of PZ_{simple} mtDNA mutations changes with continuous homeostatic turnover from simulation studies assuming a baseline copy number of 750 and absolute mutation rate of 5.0×10^{-8} per bp replication. The *x* axis shows the homeostatic turnover count and the *y* axis shows the top heteroplasmy level of postzygotic mutation.

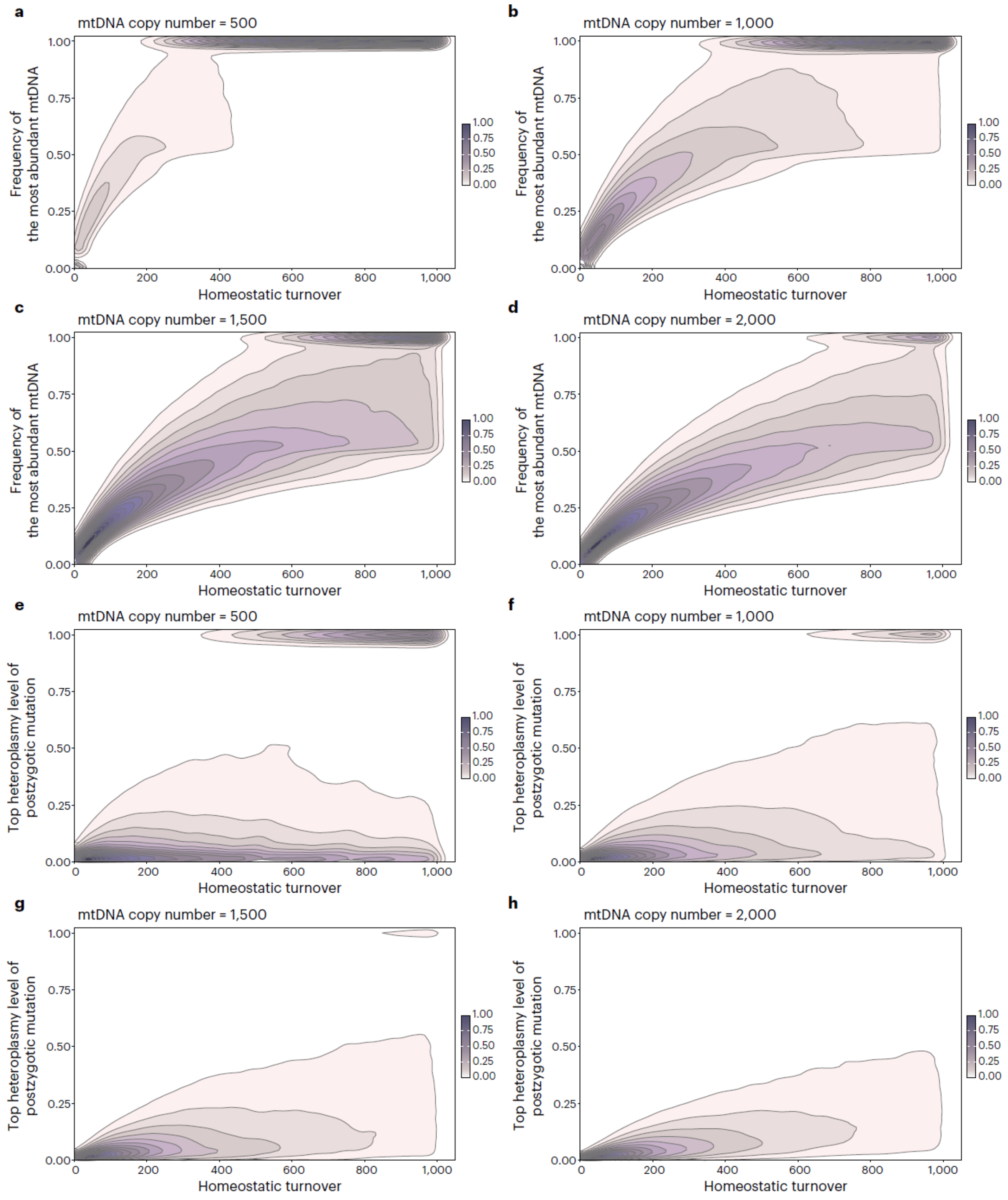

Supplementary Fig 3. Model for mtDNA dynamics with homeostatic turnover across varying mtDNA copy numbers.

a-d, Contour plots representing how an mtDNA population changes with continuous homeostatic turnover from simulation studies assuming four different baseline mtDNA copy numbers, including 500 (**a**), 1,000 (**b**), 1,500 (**c**), and 2,000 (**d**). The *x* axis shows the homeostatic turnover count and the *y* axis shows the frequency of the most prevalent mtDNA,

regardless of mutations. **e-h**, Contour plots representing how clone-VAF of PZ_{simple} mtDNA mutations changes with continuous homeostatic turnover from simulation studies assuming four different baseline mtDNA copy numbers, including 500 (**e**), 1,000 (**f**), 1,500 (**g**, and 2,000 (**h**) and absolute mutation rate of 5.0×10^{-8} per bp replication. The *x* axis shows the homeostatic turnover count and the *y* axis shows the top heteroplasmy level of postzygotic mutation.

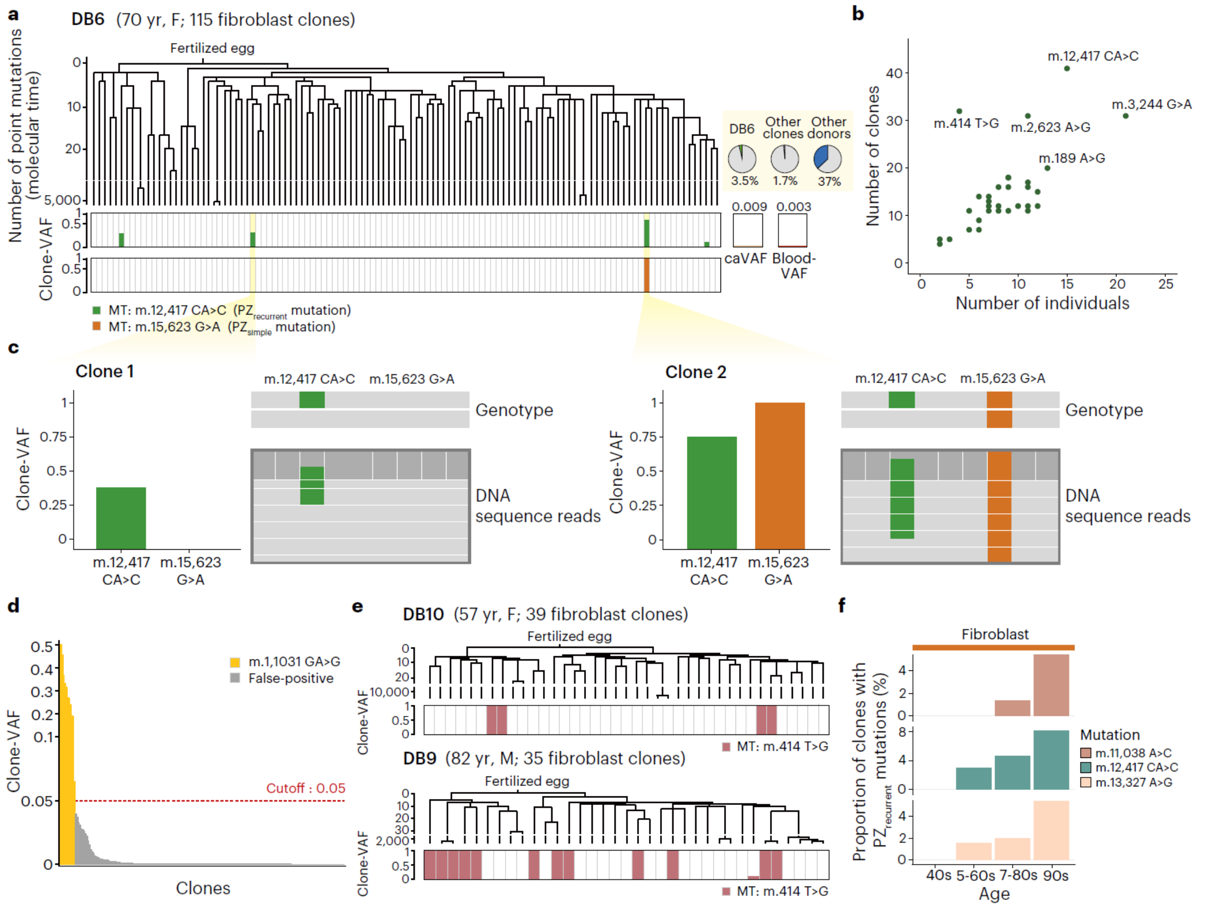

Supplementary Fig 4. Features of PZ$_{recurrent}$ mtDNA mutations.

a, Clone-VAF distributions of a PZ$_{recurrent}$ mtDNA mutation (m.12,417 CA>C) and a PZ$_{simple}$ mtDNA mutation (m.15,623 G>A) with a developmental phylogeny. The bar plots at the bottom represent clone-VAF in each clone, caVAF and blood-VAF for two alterations. Three pie charts indicate the proportions of mutant clones among clones of the individual (left), among clones of other individuals (middle), and the proportions of donors with mutant clones (right). **b,** A scatter plot illustrating the prevalence of PZ$_{recurrent}$ mutations across individuals and clones. Mutations observed in 20 or more clones are labeled. **c**, Illustrations depicting the observed VAFs and genotypes in two clones of DB6 (highlighted in yellow in **a**). Clone-VAFs, observed genotypes, and screenshots of Integrative Genomics Viewer are shown for each clone. **d**, Clone-VAF distributions of m.11,301 GA>G in 2,096 normal clones. **e**, Clone-VAFs of m.414 T>G, the PZ$_{recurrent}$ mutation, in two individuals with their developmental phylogenies. **f**, The correlation between the proportion of clones carrying PZ$_{recurrent}$ mutations and age groups.

Supplementary Table Legends

Supplementary Table 1. Summarized WGS information of individuals. The table presents summary information on each individual, including age, sex, the number of normal clones, matched bulk tissues, and matched tumor tissues.

Supplementary Table 2. Demographic and mutational characteristics of normal clones. The table presents information on each clone in the study, including mtDNA copy number and mtDNA variant count.

Supplementary Table 3. Background noise matrix of mtDNA variants. The table presents the background noise matrix on each locus and alternate allele. Mean, standard deviation, and cutoff VAF are provided.

Supplementary Table 4. Mosaic mtDNA alterations identified in this study. The table presents detailed information on each mtDNA variant identified in the study, including functional consequence, region, and variant type.

Supplementary Table 5. Fertilized egg-originated variants classified in this study. The table presents information on Het_{FE} variants, including the caVAF and clone-VAF lists.

Supplementary Table 6. Postzygotic recurrent mutations classified in this study. The table presents information on $PZ_{recurrent}$ mutations, including the number of clones, individuals, and regions.

Supplementary Table 7. Demographic and mutational characteristics of adenomas and tumors. The table presents detailed information on colorectal adenoma or cancer samples analyzed in the study, including histology information, mtDNA copy number, and mtDNA variant count.

Supplementary Table 8. Top mtDNA frequency from simulations. The table presents simulation results of the top mtDNA population frequency obtained from the mitotic turnover model (mtDNA copy number=750).

Supplementary Table 9. Top mtDNA heteroplasmy from simulations. The table presents simulation results of the top mtDNA heteroplasmy obtained from the mitotic turnover model (mtDNA copy number=750).

Supplementary References

1. Coorens, T.H.H. *et al.* Extensive phylogenies of human development inferred from somatic mutations. *Nature* **597**, 387-392 (2021).
2. Lee-Six, H. *et al.* Population dynamics of normal human blood inferred from somatic mutations. *Nature* **561**, 473-478 (2018).
3. Park, S. *et al.* Clonal dynamics in early human embryogenesis inferred from somatic mutation. *Nature* **597**, 393-397 (2021).
4. Nam, C.H. *et al.* Widespread somatic L1 retrotransposition in normal colorectal epithelium. *Nature* **617**, 540-547 (2023).
5. Lee-Six, H. *et al.* The landscape of somatic mutation in normal colorectal epithelial cells. *Nature* **574**, 532-537 (2019).
6. Griffiths, D.F., Davies, S.J., Williams, D., Williams, G.T. & Williams, E.D. Demonstration of somatic mutation and colonic crypt clonality by X-linked enzyme histochemistry. *Nature* **333**, 461-3 (1988).
7. Kozar, S. *et al.* Continuous clonal labeling reveals small numbers of functional stem cells in intestinal crypts and adenomas. *Cell Stem Cell* **13**, 626-33 (2013).
8. Kim, I.B. *et al.* Non-coding de novo mutations in chromatin interactions are implicated in autism spectrum disorder. *Mol. Psychiatry* **27**, 4680-4694 (2022).
9. Gorelick, A.N. *et al.* Respiratory complex and tissue lineage drive recurrent mutations in tumour mtDNA. *Nat Metab* **3**, 558-570 (2021).
10. Birch-Machin, M.A. & Swalwell, H. How mitochondria record the effects of UV exposure and oxidative stress using human skin as a model tissue. *Mutagenesis* **25**, 101-107 (2010).
11. Birket, M.J. & Birch-Machin, M.A. Ultraviolet radiation exposure accelerates the accumulation of the aging-dependent T414G mitochondrial DNA mutation in human skin. *Aging Cell* **6**, 557-564 (2007).
