# Supplementary material for: Mitochondrial DNA mosaicism in normal human somatic cells
Source: Nat Genet. 2024 Jul 22;56(8):1665–77. doi: 10.1038/s41588-024-01838-z (PMC11319206; doi:10.1038/s41588-024-01838-z)
Supplement: Supplementary file 2 — Reporting Summary [file 41588_2024_1838_MOESM2_ESM.pdf]

nature portfolio

Corresponding author(s): Young Seok Ju

Last updated by author(s): Jun 4, 2024

Reporting Summary

Nature Portfolio wishes to improve the reproducibility of the work that we publish. This form provides structure for consistency and transparency in reporting. For further information on Nature Portfolio policies, see our Editorial Policies and the Editorial Policy Checklist.

Statistics

For all statistical analyses, confirm that the following items are present in the figure legend, table legend, main text, or Methods section.

n/a	Confirmed	
☐	☒	The exact sample size (*n*) for each experimental group/condition, given as a discrete number and unit of measurement
☐	☒	A statement on whether measurements were taken from distinct samples or whether the same sample was measured repeatedly
☐	☒	The statistical test(s) used AND whether they are one- or two-sided<br>*Only common tests should be described solely by name; describe more complex techniques in the Methods section.*
☐	☒	A description of all covariates tested
☐	☒	A description of any assumptions or corrections, such as tests of normality and adjustment for multiple comparisons
☐	☒	A full description of the statistical parameters including central tendency (e.g. means) or other basic estimates (e.g. regression coefficient) AND variation (e.g. standard deviation) or associated estimates of uncertainty (e.g. confidence intervals)
☐	☒	For null hypothesis testing, the test statistic (e.g. *F*, *t*, *r*) with confidence intervals, effect sizes, degrees of freedom and *P* value noted<br>*Give P values as exact values whenever suitable.*
☒	☐	For Bayesian analysis, information on the choice of priors and Markov chain Monte Carlo settings
☒	☐	For hierarchical and complex designs, identification of the appropriate level for tests and full reporting of outcomes
☐	☒	Estimates of effect sizes (e.g. Cohen's *d*, Pearson's *r*), indicating how they were calculated

Our web collection on statistics for biologists contains articles on many of the points above.

Software and code

Policy information about availability of computer code

Data collection	no software was used.
Data analysis	Sequenced reads were aligned to the human reference genome (GRCh37) using BWA (v0.7.17) algorithm. The duplicated reads were removed by Picard (v2.1.0) and mitochondrial DNA sequences were extracted by Samtools (v1.10). We identified single-nucleotide variants and short indels using Varscan2 (v2.4.2) and HaplotyperCaller2 in GATK (v4.2.0.0). Detected variants were inspected using IGV (v2.11.9). The mean coverage depths of mitochondrial and nuclear genome were computed using mosdepth (v0.3.1). Custom scripts were written by Python (v3.7.0) and R (v4.1.3) and are available at GitHub (https://github.com/jisong-an/mtDNA_mosaicism).

For manuscripts utilizing custom algorithms or software that are central to the research but not yet described in published literature, software must be made available to editors and reviewers. We strongly encourage code deposition in a community repository (e.g. GitHub). See the Nature Portfolio guidelines for submitting code & software for further information.

Data

Policy information about availability of data

All manuscripts must include a data availability statement. This statement should provide the following information, where applicable:

- Accession codes, unique identifiers, or web links for publicly available datasets
- A description of any restrictions on data availability
- For clinical datasets or third party data, please ensure that the statement adheres to our policy

Whole-genome sequencing data used in the study were publicly available at the European Genome-phenome Archive (EGA) with accession no. EGAD00001007032, EGAD00001010183, EGAD00001004086, and EGAD00001007851. Whole-genome sequencing data of normal colorectal epithelium and fibroblast clones, extracted from the mitochondrial genome, are deposited in the EGA with accession no. EGAS50000000254 and available for general research use. The base substitutions and indels identified in the mitochondrial DNA are available in Supplementary Table 4. Source data are provided with this paper. The human reference genome GRCh37 is available at https://www.ncbi.nlm.nih.gov/data-hub/genome/GCF_000001405.13.

Research involving human participants, their data, or biological material

Policy information about studies with human participants or human data. See also policy information about sex, gender (identity/presentation), and sexual orientation and race, ethnicity and racism.

Reporting on sex and gender	Sex information of individuals in this study was obtained from the original papers of public dataset. The information is summarized in Supplementary Table 1. We did not find any differences in mitochondrial DNA variants between males and females. Sex and gender were not considered in study design.
Reporting on race, ethnicity, or other socially relevant groupings	Out of 31 individuals, 27 individuals are Asians and 4 individuals are Europeans. Mitochondrial germline variants and mitochondrial haplogroups differed by race, but no differences were found for other mitochondrial DNA variants. Race, ethnicity, or other socially relevant groupings were not considered in study design.
Population characteristics	Out of 31 individuals, 19 were diagnosed with colorectal cancer. The age of the individuals spanned several age groups ranging from 0 to 93. The ratio between males and females were 0.8:1.2 (14 males and 17 females).
Recruitment	One individual was an aborted fetus obtained at Bundang Seoul National University Hospital. The parents of this individual, attending the Obstetrics and Gynecology department of Bundang Seoul National University Hospital, were recruited with their consent for an infant miscarried after 12 weeks of gestation. Data for the other individuals were published previously and downloaded for this study.
Ethics oversight	All the procedures in this study were approved by the Institutional Review Board of Korea Advanced Institute of Science and Technology (approval number: KH2021-096).

Note that full information on the approval of the study protocol must also be provided in the manuscript.

Field-specific reporting

Please select the one below that is the best fit for your research. If you are not sure, read the appropriate sections before making your selection.

☒ Life sciences ☐ Behavioural & social sciences ☐ Ecological, evolutionary & environmental sciences

For a reference copy of the document with all sections, see nature.com/documents/nr-reporting-summary-flat.pdf

Life sciences study design

All studies must disclose on these points even when the disclosure is negative.

Sample size	No statistical methods were used to predetermine the sample size. We selected samples from available individuals to describe the mitochondrial DNA mosaicism in normal cells.
Data exclusions	No data were excluded from the analyses.
Replication	We used 47 pairs of mother-daughter clones (biological replicates) to estimate the rates of culture-associated mitochondrial DNA mutations. We generated 47 daughter clones by serial clonalization of ten mother clones. All attempts at replication were successful, and almost every mtDNA variant identified in mother clones was detected in daughter clones.
Randomization	Not applicable since there was no predetermined group of samples. All available samples were used.
Blinding	Not applicable since this is a descriptive study

Reporting for specific materials, systems and methods

We require information from authors about some types of materials, experimental systems and methods used in many studies. Here, indicate whether each material, system or method listed is relevant to your study. If you are not sure if a list item applies to your research, read the appropriate section before selecting a response.

Materials & experimental systems

n/a	Involved in the study
☒	☐ Antibodies
☒	☐ Eukaryotic cell lines
☒	☐ Palaeontology and archaeology
☒	☐ Animals and other organisms
☒	☐ Clinical data
☒	☐ Dual use research of concern
☒	☐ Plants

Methods

n/a	Involved in the study
☒	☐ ChIP-seq
☒	☐ Flow cytometry
☒	☐ MRI-based neuroimaging

Plants

Seed stocks

Report on the source of all seed stocks or other plant material used. If applicable, state the seed stock centre and catalogue number. If plant specimens were collected from the field, describe the collection location, date and sampling procedures.

Novel plant genotypes

Describe the methods by which all novel plant genotypes were produced. This includes those generated by transgenic approaches, gene editing, chemical/radiation-based mutagenesis and hybridization. For transgenic lines, describe the transformation method, the number of independent lines analyzed and the generation upon which experiments were performed. For gene-edited lines, describe the editor used, the endogenous sequence targeted for editing, the targeting guide RNA sequence (if applicable) and how the editor was applied.

Authentication

Describe any authentication procedures for each seed stock used or novel genotype generated. Describe any experiments used to assess the effect of a mutation and, where applicable, how potential secondary effects (e.g. second site T-DNA insertions, mosiacism, off-target gene editing) were examined.
